# A randomized clinical trial assessing theranostic-guided corneal cross-linking for treating keratoconus: the ARGO protocol

**DOI:** 10.1007/s10792-022-02628-4

**Published:** 2022-12-31

**Authors:** Anna Maria Roszkowska, Giuseppe Lombardo, Rita Mencucci, Vincenzo Scorcia, Giuseppe Giannaccare, Annarita Vestri, Danilo Alunni Fegatelli, Giuseppe Massimo Bernava, Sebastiano Serrao, Marco Lombardo

**Affiliations:** 1 Ophthalmology Department, Faculty of Medicine, Health Sciences of Andrzej Frycz Modrzewski Krakow University, Gustawa Herlinga-Grudzińskiego 1, 30-705 Krakow, Poland; 2 Dipartimento BIOMORF, Università di Messina, Via Consolare Valeria 1, 98100 Messina, Italy; 3 CNR-IPCF, Istituto per I Processi Chimico-Fisici, Viale F. Stagno D’Alcontres 37, 98158 Messina, Italy; 4 SOD Oculistica, AOU Careggi, Università di Firenze, Largo Brambilla 3, 50134 Florence, Italy; 5 UO Oculistica, AOU Mater Domini, Università Magna Graecia di Catanzaro, Viale Europa, 88100 Catanzaro, Italy; 6 Dipartimento di Sanità Pubblica e Malattie Infettive, Università di Roma “La Sapienza”, Piazzale Aldo Moro 5, 00185 Rome, Italy; 7 Studio Italiano di Oftalmologia, Via Livenza 3, 00198 Rome, Italy

**Keywords:** Corneal cross-linking, Theranostics, Riboflavin, Keratoconus

## Abstract

The *Assessment of theranostic guided riboflavin/UV-A corneal cross-linking for treatment of keratoconus* (ARGO; registration number NCT05457647) clinical trial tests the hypothesis that theranostic-guided riboflavin/UV-A corneal cross-linking (CXL) can provide predictable clinical efficacy for halting keratoconus progression, regardless of treatment protocol, i.e., either with or without epithelial removal. Theranostics is an emerging therapeutic paradigm of personalized and precision medicine that enables real-time monitoring of image-guided therapy. In this trial, the theranostic software module of a novel UV-A medical device will be validated in order to confirm its accuracy in estimating corneal cross-linking efficacy in real time. During CXL procedure, the theranostic UV-A medical device will provide the operator with an imaging biomarker, i.e., the theranostic score, which is calculated by non-invasive measurement of corneal riboflavin concentration and its UV-A light mediated photo-degradation. ARGO is a randomized multicenter clinical trial in patients aged between 18 and 40 years with progressive keratoconus aiming to validate the *theranostic score* by assessing the change of the maximum keratometry point value at 1-year postoperatively. A total of 50 participants will be stratified with allocation ratio 1:1 using a computer-generated stratification plan with blocks in two treatment protocols, such as epithelium-off or epithelium-on CXL. Following treatment, participants will be monitored for 12 months. Assessment of safety and performance of theranostic-guided corneal cross-linking treatment modality will be determined objectively by corneal tomography, corneal endothelial microscopy, visual acuity testing and slit-lamp eye examination.

## Introduction

Keratoconus (KC) is a naturally occurring eye disease characterized by progressive thinning and steepening of the cornea, resulting in corneal optical distortion with irregular astigmatism, increasing myopia and, at later stages, corneal opacity. Disease prevalence is about 1% with difference among geographical locations (e.g., higher in the Mediterranean and Middle East areas than northern Europe); KC still represents the primary cause of corneal transplantation in young adults globally. The early onset and the frequent progression toward vision loss contribute to the significant social and economic burden of the disease. [1–4].

Riboflavin/UV-A corneal cross-linking (CXL) is a well-established procedure used to slown down or halt disease progression; it has been introduced in Europe in 2003, thereafter it has been adopted in eastern countries and United States. Several CXL protocols, which differ in the type and time of riboflavin application and UV-A irradiance treatment settings, have been clinically validated for treatment of keratoconus. The former CXL protocol, also known as *Dresden protocol*, consists in removing the epithelium and administering a dextran-enriched riboflavin ophthalmic solution onto the corneal stroma for 30 min; afterwards the cornea is irradiated by a UV-A light device using 3 mW/cm^2^ power density for 30 min, with a total delivered energy density of 5.4 J/cm^2^. During the last decade, several UV-A light irradiation treatment protocols have been developed and clinically validated, including the most commonly used in Europe, which consists of 10 mW/cm^2^ (or 9 mW/cm^2^) UV-A power density for 9 min (or 10 min), with a total delivered energy density of 5.4 J/cm [2, 5, 6].

In the same period, dextran-free riboflavin ophthalmic solutions have been increasingly used based upon pre-clinical and clinical evidences on their higher benefit/safety profile in comparison with dextran-enriched ophthalmic solution for the indication of use [7–9].

CXL treatment proved to be a valid therapy in reducing the need for keratoplasty for thousands patients affected by KC [10]; however, according to the scientific evidence, the procedure shows a huge variation in efficacy (where efficacy has been determined as stabilization or flattening of the maximum keratometry point value, *K*_max_), with a success rate ranging from 10 to 90% 12 months after surgery [11–16]. Epithelial removal is considered a fundamental pre-requisite to improve therapeutic efficacy; nevertheless, it represents the predisposing factor for the most frequent and major complications of the epithelium-off protocol, which include in virtually all cases ocular pain, transient corneal edema and corneal haze, while in some cases severe adverse events, such as corneal infections, corneal melting and corneal scarring, which cause vision loss [15–17]. An additional risk factor in the epithelium-off protocol is the treatment of corneal tissues with a central thickness lower than 400 μm (with intact epithelium), due to the risk of inducing phototoxic damage to corneal endothelial structures [18]. For this reason, any technical improvement, which can improve efficacy and minimize risks of the epithelium-off technique, is highly desirable. Although several transepithelial, or epithelium-on, treatment protocols have been developed in order to minimize these risks, their clinical efficacy still remains object of debate [19–23]. Currently, transepithelial CXL treatment protocol remains challenging since it lacks of selectivity in understanding the amount of the therapeutic molecule penetrating into the corneal stroma through the intact epithelium.

The precise knowledge about the principles of UV-A light/riboflavin interaction with the cornea could be fundamental to advance the therapeutic management of keratoconus with CXL [24–26]. In the presence of UV-A light, riboflavin exhibits photosensitizing properties, reacting with a wide range of electron-donating substrates or even in the absence of added electron donor, through mixed Type I and Type II photochemical mechanisms [27–31]. The main mechanism of the riboflavin/UV-A CXL procedure has been shown to consist in the direct interaction between riboflavin triplets and reactive groups of stromal proteins, which leads to the cross-linking of the proteins through radical reactions [32]. This implies that, in ambient environment, the amount of riboflavin into the cornea and the role of Type I mechanism are predominant for the formation of additional chemical bonds between stromal proteins [22, 30].

Theranostics is an emerging and breakthrough therapeutic paradigm of personalized and precision medicine; the term refers to the simultaneous integration of therapy and diagnostics. Integrating theranostics technology with advanced UV-A device for CXL procedure allows for tailoring the precise therapeutical dose of riboflavin and its UV-A light photo-activation to the individual cornea. This novel approach, through real-time monitoring of corneal riboflavin concentration, has the scope to improve outcome predictability and to minimize risks of adverse events on a personal basis. Recently, a theranostic UV-A medical device has been made available for treating keratoconus by theranostic-guided corneal cross-linking. Pre-clinical studies have provided enough evidence on accuracy and precision of the theranostic UV-A medical device for inducing highly predictable tissue stiffening in human donor corneal tissues [33–36]. The novel UV-A device has emerged as a promising and powerful tool to precisely monitor the diffusion of riboflavin into the corneal stroma and its UV-A light mediated photo-degradation during treatment. In addition, it holds the ability to estimate CXL treatment efficacy providing an imaging biomarker, such as the *theranostic score*, which correlates with the treatment-induced stromal stiffening effect [33, 36].

This manuscript describes the design of the clinical study entitled “*Assessment of theranostic guided riboflavin/UV-A corneal cross-linking for treatment of keratoconus* “, whose acronym is ARGO. The scope of the trial is to validate the theranostics software module of the novel UV-A medical device (C4V CHROMO4VIS™ v. 2.0); the primary outcome measure of the study consists in assessing the predictive ability of the *theranostic score* in assessing clinical efficacy of the corneal cross-linking procedure.

## Methods and analysis

### Study objective

The scope of the study is to evaluate the novel modality of theranostic based riboflavin/UV-A corneal cross-linking based on theranostics aiming at improving treatment predictability for better eye care to patients suffering from KC. The objective of the study is to validate the *theranostic score* by assessing the change of corneal topography *K*_max_ value at 12 months after riboflavin/UV-A corneal cross-linking for the treatment of KC.

### Study design

ARGO is a randomized clinical trial conducted in three University centers in Italy (University of Catanzaro, University of Florence and University of Messina). Patients with a confirmed diagnosis of progressive KC are evaluated for suitability as candidates for CXL.

The trial consists of one study arm receiving riboflavin/UV-A CXL with either epithelium-off (Epi-OFF) or epithelium-on (Epi-ON) treatment protocol. Only 1 eye of each participant is designated as the study eye; if both eyes of a participant are eligible, the eye with lower corrected distance visual acuity (CDVA) is chosen as the study eye.

Before any test is conducted, the participant is given a brief explanation of the study (including the number and time of visits to be performed); informed consent is obtained from each participant before randomization and performance of the CXL procedure. Participants are evaluated at baseline, day 0 (treatment), day 7, day 30, day 90, day 180 and day 360 after treatment.

The study design assumes that the theranostic based riboflavin/UV-A corneal cross-linking treatment does not induce in the participants unacceptable risks. Several clinical studies have shown safety and efficacy of CXL procedure for the indication of treating patients with keratoconus [17, 37–41]. The occurrence of severe adverse events, such as ulcerative keratitis or corneal melting, have been registered in less than 0.5% Epi-OFF CXL procedures and no severe adverse events have been recorded after Epi-ON CXL procedures.

### Ethical and safety considerations

The clinical study adheres with the ethical principles that have their origin in the Declaration of Helsinki, the Convention of Oviedo and is consistent with GCP, including the ICH Guideline for good clinical practice E6, the Directive 2001/20/EC, the ISO 14155:2011, the Regulation (EU) 536/2014 of the European Parliament and of the Council of 16 April 2014, the Regulation (EU) 2017/745 of the European Parliament and of the Council of 5 April 2017, the Ministerial decree of the Italian Minister of Health of 2 August 2005, the Decree n. 211 of 24 June 2003 and the Ministerial Decree of 14 July 2009.

The study is registered at clinicaltrials.gov with registration number NCT05457647 (available at https://clinicaltrials.gov/ct2/show/NCT05457647?term=NCT05457647&draw=2&rank=1); it was approved by the Italian Ministry of Health (Prot. N. DGDMF/I.5.i.m.2/2021/2024 05/01/2022) and ethical approval was granted by the Ethics Committees of Regione Calabria Sezione Area Centro (Prot. N. 358 18/11/2021), AOU Gaetano Martino (Prot. N. 101 23/11/2021) and Area Vasto Centro (Prot. N. 21250_spe 18/01/2022).

### Eligibility criteria and definition of disease progression

According to the medical literature, [42] the criterion to determine progression of KC was based on providing at least one of the following conditions:

- at least two Placido disk corneal topography measurements showing at least + 1.00 D steepening of the *K*_max_ value in the last year;
- at least two manifest refraction measurements showing at least − 0.50 D change in spherical equivalent refraction in the last year;
- at least two central corneal thickness (CCT) measurements showing at least − 10 µm change in in the last year.

The exclusion criteria were corneal apex steeper than 63 D, corneal thickness thinner than 400 µm; corneal scarring; descemetocele; history of herpetic keratitis; concomitant eye diseases; inflammatory eye diseases; glaucoma; cataract; nystagmus; pregnancy; breast feeding.

### Stratification and allocation of participants to treatment groups

The study consists of a study arm receiving either investigational theranostic-guided epithelium-off corneal cross-linking treatment or investigational theranostic-guided transepithelial (epithelium-on) corneal cross-linking treatment. Eligible participants are stratified with allocation ratio 1:1 into either treatment protocol using a computer-generated stratification plan with blocks. Two different blocks are created, which include eyes with *K*_max_ steeper or flatter than 54.0 D to allocate patients with comparable baseline *K*_max_ values in either treatment protocol. The stratification code is given to each local site of investigation by the central monitoring site of the Sponsor after the participant has been considered eligible to the study and has signed the informed consent. The enrolment is competitive.

### Baseline assessment

Potential candidates undergo a complete eye examination to determine their eligibility to the study. The baseline visit (visit 1) provides the confirmation that the participant is eligible for the inclusion in the study and the full medical history is collected. Baseline measurements are performed in the following order:

1. corneal curvature and corneal thickness measurements;
2. endothelial cell density (ECD) measurement;
3. uncorrected distance visual acuity (UDVA);
4. corrected distance visual acuity (CDVA);
5. manifest spherical equivalent refraction (MSER);
6. slit lamp bio-microscopy of the ocular surface and the anterior segment of the eye;
7. intra-ocular pressure (IOP) measurement;
8. dilated fundus examination.

At the end of the baseline assessment, eligible participants must sign the informed consent form before to be assigned an individual stratification number according to the stratification protocol described above.

### Intervention: theranostic UV-A medical device

The C4V CHROMO4VIS™ (Regensight srl, Rome, Italy) is a portable electronic medical device, which delivers ultraviolet light (365 nm wavelength) in a homogeneous circular spot onto the area of the cornea to treat after a riboflavin ophthalmic solution has been properly applied. Emitted UV-A irradiance and energy dose are continuously controlled by an on-board computer system during operation. The medical device consists of an articulating arm, a serving tray to support medical and surgical supplies items and a wheeled cart.

The optics head of the UV-A device houses the electronic boards, the light sources, the iris aperture, the camera and the Placido disk, which is used to precisely focus the UV-A light beam onto the cornea to treat. The medical device has undergone safety testing according to the EMC EN 60601-1-2, EN 60601-1, ISO 15004-2:2007 and EN 64271:2010 standards; the performance of the UV-A medical device in question has been validated in laboratory studies, which have shown that the *theranostic score* had excellent accuracy and precision in predicting the tissue biomechanical strengthening induced by corneal cross-linking procedure in eye bank human donor tissues [33–36].

Before UV-A irradiation, the cornea is soaked with riboflavin. In this trial, a CE certified hypotonic, dextran-free, 0.22% riboflavin ophthalmic solution (Ritsight™, Regensight srl, Rome, Italy) is used in all cases. The ophthalmic solution has been formulated to allow the effective penetration of riboflavin into the corneal stroma, even with the epithelium intact. The riboflavin ophthalmic solution has undergone biocompatibility testing, including citotoxicity by direct contact test (ISO 10993-5:2009), acute ocular irritation test (ISO 10993-10:2010) and delayed hypersensitivity test (ISO 10993-10:2010).

Table 1 summarizes the main parameters of the investigational theranostic UV-A medical device and the riboflavin ophthalmic solution used in the clinical study.

**Table 1:** Investigational theranostic UV-A device and riboflavin ophthalmic solution used in ARGO trial

Theranostic UV-A device: C4V CHROMO4VIS™ v. 2.0	Riboflavin ophthalmic solution: RitSight™
- Focusing mode:	– Main ingredient: riboflavin sodium phosphate
• Placido disk with augmented reality	– Composition: 0.31% (equivalent to 0.22% free base riboflavin)
- Modalities of riboflavin application:	
• Manual	
• Active	
- Modalities of UV-A light irradiation:	
• Continuous mode	
▪ UV-A power density range: 3–36 mW/cm^2^ (up to 40 mW/cm^2^ in “Enhanced EpiOn” option*)	
▪ UV Irradiation Time: 2–30 min	
UV Energy: 3.6–10.0 J/cm^2^	
• Pulsed mode	
▪ UV Power: 3–36 mW/cm^2^ (up to 40 mW/cm^2^ in “Enhanced EpiOn” option*)	
▪ UV irradiation time: 4–24 min	
▪ UV ON time: 1.0 s	
▪ UV OFF time: 1.0 s	
▪ UV Energy: 3.6–10.0 J/cm^2^	

*The treatment option “Enhanced EpiOn” is enabled if the Epi-On protocol is selected by the user. By selecting the “Enhanced EpiOn” option, the UV energy dose is increased by 10% to compensate for the epithelium UV absorbance property

#### Intervention: investigational theranostic-guided CXL

Theranostic-guided corneal cross-linking consists of two main phases, each providing the operator with real time quantitative information on performance of CXL procedure. The first phase, during application of riboflavin, includes light-mediated measures of the corneal riboflavin concentration providing the operator with an estimation of this parameter in real time; the riboflavin dose is calculated into the targeted area of the cornea by imaging the fluorescence emitted from the tissue soaked with the photo-sensitizing molecule. In the latter phase (UV-A light photo-therapy), the UV-A light is used both for quantitative imaging and therapy; during this phase, the UV-A device computes at run-time a *theranostic score*, which takes into account the corneal riboflavin concentration achieved prior to starting UV-A photo-therapy phase. The *theranostic score* has been validated in pre-clinical studies showing to highly correlate with increased tissue stiffening induced by CXL procedure, thus highlighting its potential of for real time prediction of CXL treatment efficacy in vivo [33–36].

In this clinical study, the CXL procedure is performed using the theranostic software module of the C4V CHROMO4VIS™ device (sw v. 2.0) in all participants. Participants receive a single dose of the 0.22% riboflavin ophthalmic solution. Application of the riboflavin eye drop is done for 15 min in the Epi-OFF CXL treatment protocol and for 20 min in the Epi-ON CXL protocol. Corneal riboflavin concentration is estimated by the C4V CHROMO4VIS™ system during the dosing phase of treatment. Once the dosing phase is completed, the cornea is irradiated by 10 mW/cm^2^ UV-A power density for 9 min (5.4 J/cm^2^ energy density) with 7.00 mm irradiation beam diameter in all participants. During UV-A irradiation, the theranostic UV-A device monitors the UV-A light induced riboflavin photo-degradation and estimates treatment efficacy providing a *theranostic score* to the operator.

It is important to note that in this trial, the theranostic UV-A device monitors the clinical parameters of the cornea (i.e., riboflavin dose and *theranostic score* correlating with tissue stiffening) during operation and does not provide preferred outcome measures to the operator (e.g., it does not alert the operator to stop or continue riboflavin dosing until the pre-set time is completed). In addition, the investigational treatment settings cannot be changed by operators. The scope is to validate the *theranostic score* without introducing further treatment variables other than those of the patient’s cornea to treat. Treatment settings are pre-set for both treatment protocols that are object of investigation in the present clinical study, as shown in Table 2.

**Table 2:** Pre-specified theranostic-guided study protocol settings of the ARGO trial

Investigational theranostic-guided Epi-OFF protocol	Investigational theranostic-guided Epi-ON protocol
Riboflavin dosing mode: manual	Riboflavin dosing mode: manual
Riboflavin dosing time: 15 min	Riboflavin dosing time: 20 min
UV-A irradiation mode: continuous	UV-A irradiation mode: continuous
UV-A irradiation power: 10 mW/cm^2^	UV-A irradiation power: 10 mW/cm^2^
UV-A irradiation time: 9 min	UV-A irradiation time: 9 min
UV-A energy dose: 5.4 J/cm^2^	UV-A energy dose: 5.4 J/cm^2^
Beam aperture: 7 mm	Beam aperture: 7 mm

The investigational riboflavin/UV-A CXL protocol is described as follows:

- before starting treatment, the operator scans the data matrix code of the riboflavin ophthalmic solution using the video traceability wizard system of the theranostic UV-A medical device;
- the operator adds the key code of the eligible patient to be treated in the “patient database screen” of the system and further adds the eye to be treated (Right or Left), the corneal parameters (CCT and *K*_max_) and manifest refraction data (sphere, cylinder, axis) in the “treatment plan screen” of the system;
- the operator selects the pre-specified treatment protocol settings for the patient and confirms to begin treatment;
- upon preparation of the patient’s eye for treatment, the operator applies the riboflavin ophthalmic solution one drop every 20 s directly onto the cornea of the eye to treat for the pre-set dosing time;
- at pre-specified intervals during dosing phase (total four measurements), the system alerts the operator to press the footswitch for measuring the concentration of riboflavin into the cornea. The operator is warned to invite the patient to fixate to the fixation light of the system and to eventually remove the excess of riboflavin onto the cornea before pressing the footswitch;
- the C4V CHROMO4VIS™ system provides an estimate of the riboflavin concentration, tracks the riboflavin application time and notifies the operator once the induction is complete;
- the operator selects to proceed to UV-A light irradiation of the cornea by pressing the footswitch;
- at pre-specified intervals during UV-A irradiation phase (total four measurements), the system alerts the operator that is measuring riboflavin concentration to estimate treatment efficacy by calculating the *theranostic score*;
- the operator may choose to rinse with balanced salt solution the corneal surface at 3 min interval during UV-A irradiation;
- the C4V CHROMO4VIS™ system tracks the UV-A treatment time, turns off the UV-A light and notifies the user when the treatment has been completed;
- at the end of treatment, a report including the anonymized treatment data is generated by the system; the anonymized report is uploaded—through remote access—to the central monitoring site.

**Fig. 1:**
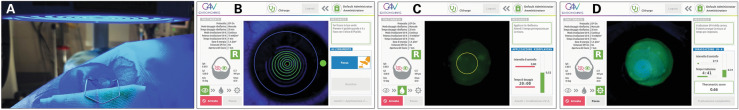
Main treatment steps of investigational theranostic-guided CXL procedure. **A** The patient lies supine looking at the fixation light of the system. The UV-A device drives the operator through the main procedure steps of theranostic-guided CXL, inviting her/him to proceed to the next phase by pressing the footswitch; **B** focusing phase; **C** dosing phase and **D** UV-A irradiation phase. The Placido disk of the system allows for a precise focusing of the UV-A beam onto the area of the cornea to treat. At pre-specified intervals during dosing and UV-A photo-therapy phases, the system tracks the concentration of riboflavin into the cornea and provides the operator with real time quantitative information on treatment performance. The *theranostic score* provides an estimation of treatment efficacy

**Fig. 2:**
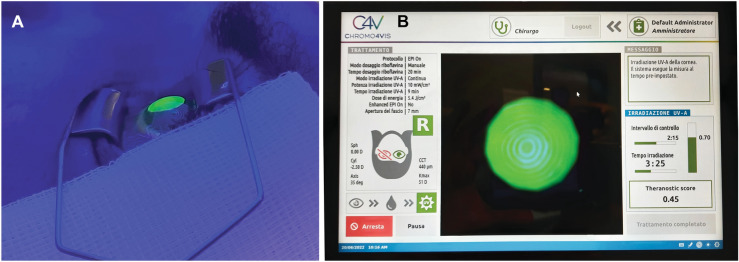
During theranostic-guided UV-A irradiation of the cornea enriched with riboflavin, the system calculates the *theranostic score*. In **A**, **B** a representative case of epithelium-on investigational theranostic-guided CXL procedure. The theranostic medical device is able to quantify the amount of UV-A light mediated photo-degradation of riboflavin providing a real time estimate (i.e., *theranostic score*) that correlates with the induced corneal stiffening effect of CXL. Calculation of the *theranostic score* takes into account the corneal riboflavin dose prior to start UV-A photo-therapy phase, the amount of riboflavin photo-degraded by UV-A light therapy and the corneal thickness

Figure 1 shows the main treatment steps of investigational theranostic-guided CXL procedures; Fig. 2 shows the treatment area and the real time corneal riboflavin concentration monitoring performed by the theranostic UV-A device.

#### Study assessment

This is an observer-masked clinical trial; the Case Report Form (CRF) is anonymized including only the stratification code of the participant. After treatment, each participant is assessed at day 7 (visit 2), day 30 (visit 3), day 90 (visit 4), day 180 (visit 5) and day 360 (visit 6). These visits will provide follow-up measurements, which will be performed in the following order:

1. Corneal curvature and corneal thickness measurements using the computerized corneal topography/pachymetry device.
2. ECD measurements using the specular microscopy.
3. UDVA measured with ETDRS chart at a test distance of 4 m.
4. CDVA and MSER measured with ETDRS chart at a test distance of 4 m.
5. Slit lamp bio-microscopy of the ocular surface and the anterior segment of the eye.

A ± 2 working day tolerance window will be allowed for visit 2; a ± 4 days tolerance window will be allowed for visit 3; a ± 10 days tolerance window will be allowed for visit 4 and a ± 14 days tolerance window will be allowed for visit 5. A ± 21 working days tolerance window will be allowed for visit 6, which will be the last study visit for all participants enrolled in the study.

A tabulated summary of the study visits is shown in Table 3.

**Table 3:** Scheduled ARGO trial visits

Time	Baseline	Day 0	Day 7	Day 30	Day 90	Day 180	Day 360
Description*	Visit 1	Treatment	Visit 2	Visit 3	Visit 4	Visit 5	Visit 6
Informed consent	×						
Medical history	×						
UDVA	×		×	×	×	×	×
CDVA	×		×	×	×	×	×
MSER	×		×	×	×	×	×
Corneal Topography/pachymetry	×		×	×	×	×	×
Corneal specular microscopy	×		×	×	×	×	×
Slit lamp microscopy	×		×	×	×	×	×
Ocular tonometry	×						×
Dilated fundus examination	×						×
Theranostic data		×					
Adverse events		×	×	×	×	×	×

*UDVA Uncorrected distance visual acuity, *CDVA* corrected distance visual acuity, *MSER* manifest spherical equivalent refraction

#### Outcome measures

##### Primary outcome measure: validation of the theranostic score [Time Frame: 12-months]

- ROC curve analysis is used to evaluate cut-off values for the *theranostic score* in predicting the propensity of maximum keratometry point value (defined as − 1.05 D; see “sample size calculation” section).

Confirmation of the *theranostic score* is provided at the end of treatment procedure. Study success is defined as detecting a difference of 0.2500 between the area under the ROC curve (AUC) under the null hypothesis of 0.6000 and an AUC under the alternative hypothesis of 0.8500 using a two-sided *z*-test at a significance level of 0.05000.

##### Secondary outcome measures: efficacy [Time Frame: 12-months]

Efficacy data will be identified by measurement of corneal curvature with computerized topography in order to assess changes of the *K*_max_ value (D) after treatment.

- Change of *K*_max_ value assessed by corneal topography at 12-months.

The investigator records the *K*_max_ value at each visit and fills the corresponding CRF. This test is performed to provide evidence of corneal curvature change after treatment.

##### Secondary outcome measures: safety [Time Frame: 12-months]

Safety data will be identified by using corneal specular microscopy.

- Change of ECD assessed by specular microscopy at 12-months.

The investigator records the ECD (cell/mm^2^) count at each visit and fills the corresponding CRF. This test is performed to provide evidence of corneal cells integrity after treatment.

##### Other outcome measures

- Change of MSER assessed by ETDRS at 12-months.

MSER is determined using a standard ETDRS chart under photopic conditions (with the luminance of the test at 85 cd/m^2^) at a test distance of 4 m. The manifest refraction is assessed by using trial lenses and is expressed in diopters (D). This test is performed to provide evidence of optical focusing properties change after treatment.

- Change of CDVA at 12-months.

CDVA is determined using a standard “Early Treatment Diabetic Retinopathy Study” (ETDRS) chart under photopic conditions (with the luminance of the test at 85 cd/m^2^) at a test distance of 4 m. The CDVA is expressed in LogMAR. The patient is scored by the LogMAR of the line with ≥ 3 letters he/she is able to read.

- Change of Central Corneal Thickness assessed by corneal pachymetry at 12-months.

Measurement of central corneal thickness (CCT, µm) is done by using corneal tomography. The investigator records the CCT value at each visit and must fill the corresponding CRF. This test is performed to provide evidence of corneal thickness change after treatment.

- Interim assessment of the *theranostic score* [Time Frame: 6-months]

ROC curve analysis is used to evaluate cut-off values for the theranostic score in predicting the propensity of Maximum Keratometry (defined as − 0.79 D; see “Methodology for sample size calculation” section) at 6-months postoperatively.

**Fig. 3:**
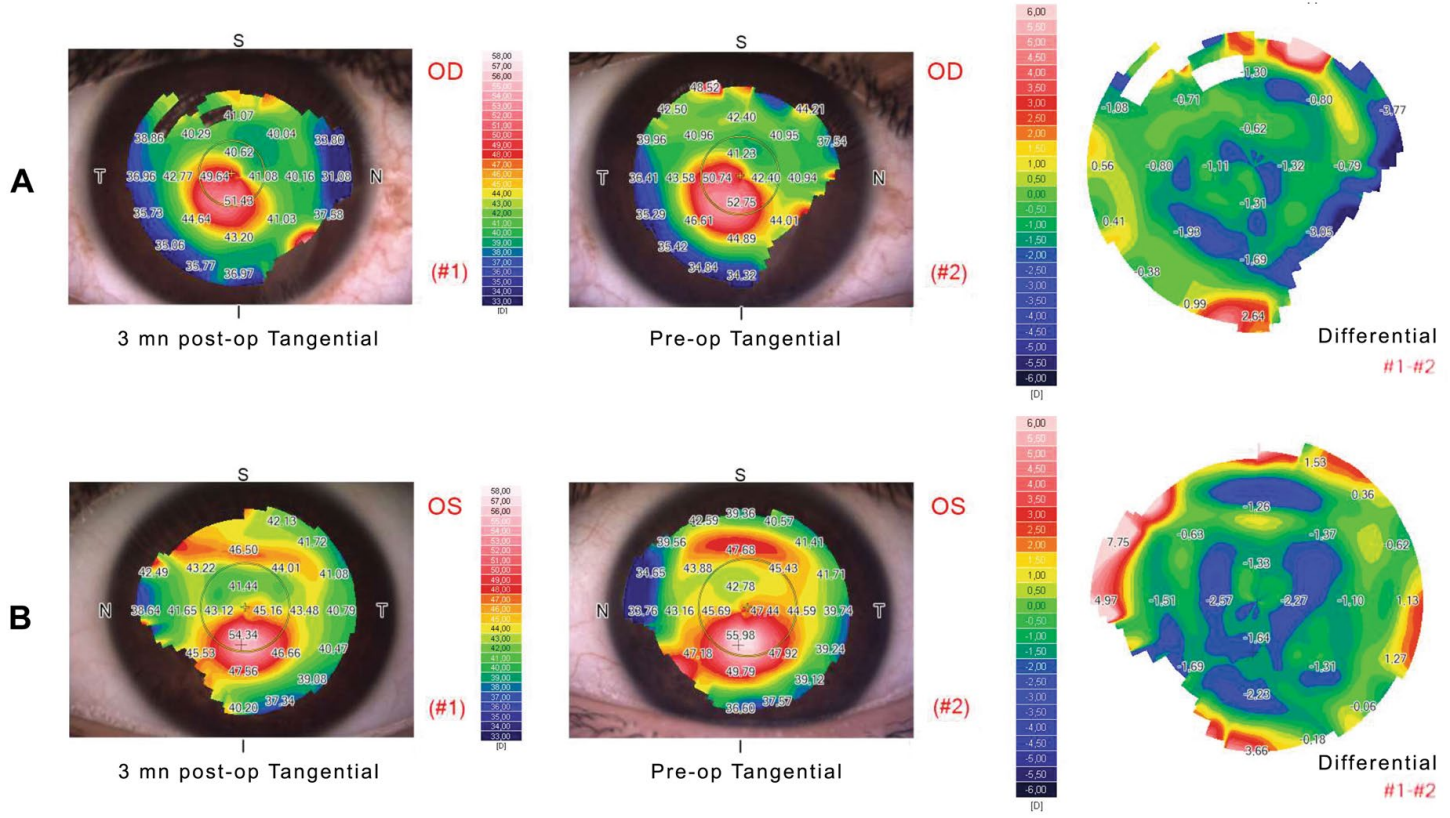
Representative Placido disc corneal topographies of two study cases (AOF01 and AON03) treated by investigational theranostic-guided Epi-OFF (A) and Epi-ON (B) CXL procedures respectively. The tangential maps at 3 months postoperatively and at the preoperative state are shown in (#1) and (#2) respectively. The difference tangential maps show − 1.31 D and − 1.64 D *K*_max_ flattening after Epi-OFF and Epi-ON CXL protocols respectively. The *theranostic score* was 0.97 and 0.99 respectively. “OD” and “OS” indicate “right eye” and “left eye” respectively

Figure 3 depicts the 3 months follow-up corneal topography changes in two representative study cases.

#### Adverse events

An adverse event (AE) is defined as any untoward medical occurrence in a participant enrolled into a clinical trial regardless of its causal relationship to study treatment. Adverse events are assessed from the time the participant receives the treatment until exit from the study. All AEs that occur during the study must be reported in detail on the CRF according to the ICH Guidelines and the MDCG 2020-10-2—Guidance safety report form. The Medical Dictionary for Regulatory Activities (MedDRA^®^) is used to code all AEs, which will be followed to satisfactory resolution or until the principal investigator of the Site of Investigation deems the event to be chronic or the participant to be stable. The description of the AE in the CRF must include the onset date, duration, date of resolution, severity, seriousness, etiology, and the likelihood of relationship of the AE to study treatment.

#### Sample size calculation

By expecting a response rate of 50% of cases that will reach the threshold of -1.05 D of the *K*_max_ value, a sample size of 42 patients achieves 91% power to detect a difference of 0.250 between the area under the ROC curve (AUC) under the null hypothesis of 0.6000 and an AUC under the alternative hypothesis of 0.8500 using a two-sided z-test at a significance level of 0.050. The data are continuous responses. Considering a ≤ 20% drop-out rate (determined from systematic literature review on controlled and open clinical studies on CXL procedures), a number of 50 participants (25 per treatment protocol) is allowed to be enrolled in the study.

#### Methodology for sample size calculation

Sample size calculation has been based on a thorough literature search on clinical investigations of the state of the art on corneal cross-linking for the treatment of KC.

The inclusion criteria of the literature search included the following parameters:

A. Population: patients affected by keratoconus.
B. Intervention: corneal cross-linking.

Outcome/endpoint: change of *K*_max_ value measured with corneal topography.

The exclusion criteria included the following parameters:

C. lack to report adequate information on study methodology (population, intervention, statistics);
D. follow-up shorter than 1-year follow-up after corneal cross-linking.
E. present case report or small (< 15 participants) case series or have been published as a letter to the Editor;
F. are retrospective clinical studies;
G. have not been published or have been published in scientific journals without impact factor or in journal with impact factor lower than 1.5 (i.e., the last quartile of the JcR category “Ophthalmology” in 2018);
H. have been published earlier than 2013.

The search output provided n.17 publications [11, 12, 19, 20, 40, 43–54]. The analysis of pertinent data from 6.995 cases has shown an average *K*_max_ change of − 1.05 ± 0.80 D at 1 year follow-up visit after CXL treatment. The average *K*_max_ change was − 0.79 ± 0.75 D at 6-month follow-up.

A further literature search was done to understand whether the CXL treatment protocol (i.e., Epi-OFF or Epi-ON) may influence the change of *K*_max_ value at 1-year follow-up.

The inclusion criteria of the literature search included the following parameters:

A. Population: patients affected by keratoconus.
B. Intervention: corneal cross-linking.
C. Type of study: randomized controlled trial.

Outcome/endpoint: change of maximum keratometry (*K*_max_) measured with corneal topography.

The exclusion criteria included the following parameters:

D. lack to report adequate information on study methodology (population, intervention, statistics);
E. have follow-up shorter than 1-year follow-up after corneal cross-linking;
F. present case report or small (< 15 participants) case series or have been published as a letter to the Editor;
G. are retrospective clinical studies;
H. are meta-analysis studies;
I. have not been published or have been published in scientific journals without impact factor or in journal with impact factor lower than 1.5 (i.e., the last quartile of the JcR category “Ophthalmology” in 2018);
J. have been published earlier than 2013.

The search output provided n.6 publications [44, 45, 55–59]. The analysis of pertinent data from the above articles provided the following results:

- n. 354 cases have been treated by Epi-OFF corneal cross-linking showing an average *K*_max_ change of -1.41 ± 1.71 D at the 1 year follow-up visit; the average *K*_max_ change was − 0.89 ± 0.60 D at the 6-month follow-up visit;
- n. 91 cases have been treated by transepithelial corneal cross-linking showing an average *K*_max_ change of − 0.32 ± 1.55 D at the 1 year follow-up visit; the average *K*_max_ change was -0.52 ± 1.20 D at the 6-month follow-up visit;
- in n. 122 control, untreated cases, the natural history of keratoconus progression has shown a mean change of *K*_max_ + 0.89 ± 2.70 D during 1-year follow-up; the average *K*_max_ change was + 0.93 ± 1.35 D at 6-month follow-up.

#### Statistical analysis plan

Descriptive statistics will be used to summarize the data: numerical variables will be summarized as mean and standard deviation or median and interquartile range, respectively, according to the distribution of the data; categorical variables will be represented as frequencies and proportions. Student’s t-test or Wilcoxon test will be applied to compare continuous variables, while Fisher's exact test or Chi-Squared test will be used to analyze categorical variables.

ROC curve analysis will be used to evaluate cut-off values for the *theranostic score* in predicting the propensity of *K*_max_ value. The cut-off value for the biomarker score will be determined by optimizing the Youden index. Moreover, sensitivity, specificity, positive predictive values (PPV) and negative predictive values (NPV), and confidence intervals will be calculated. Finally, the predictive ability of the final models will be determined using cross-validation methods.

At each time point, Pearson and Spearman correlation will be used to describe the relationship between numerical variables. Mixed effect models will be assessed to evaluate the longitudinal effect of the *theranostic score* on the *K*_max_ and ECD values.

Statistical significance will be set at 0.05. All the analysis will be performed using the statistical software R (latest version available).

The analysis will be conducted following the Intent-to-Treat (ITT) and the Per Protocol (PP) principles. The ITT Population consists of all participants who are enrolled into the trial and performed at least 1 follow-up visit. The PP Population will be a subset of the ITT population and will consists of all participants who participated and completed at least 66% of all the visits (i.e., four of six total visits) and the last visit 6 at day-360 and in whom there are no major protocol deviations.

## Conclusion

The ARGO trial is expected to open a new frontier in the management of patients affected by KC. Once the accuracy of the *theranostic score* in predicting CXL treatment efficacy is confirmed, the theranostic software module of the UV-A device will be fully activated and ready for assisting surgeons to tailor treatment of KC to individual patients with the highest benefit/safety profile. Since the theranostic UV-A device incorporates a machine learning algorithm, the increasing use by surgeons is further expected to provide additional features improving its performance with time, allowing the operator to select CXL treatment settings on individual basis.

Targeting the primary outcome of the ARGO trial will be especially important to solve the limits of transepithelial CXL, caused by unknown penetration of riboflavin into the stroma and possible influence of the intact epithelium in filtering UV-A light and decreasing riboflavin photo-activation. The clinical outcome of the trial is expected to answer these questions, thus advancing CXL therapy to an unprecedented level for the best attainable management of KC.
